# Fecal microbiota dynamics and its relationship to diarrhea and health in dairy calves

**DOI:** 10.1186/s40104-022-00758-4

**Published:** 2022-10-28

**Authors:** Hongwei Chen, Yalu Liu, Kailang Huang, Bin Yang, Yuanyuan Zhang, Zhongtang Yu, Jiakun Wang

**Affiliations:** 1 Institute of Dairy Science, College of Animal Sciences, Zhejiang University, Hangzhou, China; 2 MoE Key Laboratory of Molecular Animal Nutrition, Zhejiang University, Hangzhou, China; 3 Department of Animal Sciences, The Ohio State University, Columbus, OH USA

**Keywords:** Calf diarrhea, Co-occurrence pattern, Dynamic development, Fecal microbiota

## Abstract

**Background:**

Diarrhea is a major cause of morbidity and mortality in young calves, resulting in considerable economic loss for dairy farms. To determine if some gut microbes might have resistance to dysbiotic process with calf diarrhea by dictating the microbial co-occurrence patterns from birth to post-weaning, we examined the dynamic development of the gut microbiota and diarrhea status using two animal trials, with the first trial having 14 Holstein dairy calves whose fecal samples were collected 18 times over 78 d from birth to 15 d post-weaning and the second trial having 43 Holstein dairy calves whose fecal samples were collected daily from 8 to 18 days of age corresponding to the first diarrhea peak of trial 1.

**Results:**

Metataxonomic analysis of the fecal microbiota showed that the development of gut microbiota had three age periods with birth and weaning as the separatrices. Two diarrhea peaks were observed during the transition of the three age periods. Fusobacteriaceae was identified as a diarrhea-associated taxon both in the early stage and during weaning, and *Clostridium*_*sensu_stricto_1* was another increased genus among diarrheic calves in the early stage. In the neonatal calves, *Prevotella*_*2* (ASV4 and ASV26), *Prevotella*_*9* (ASV43), and *Alloprevotella* (ASV14) were negatively associated with *Clostridium_sensu_stricto_1* (ASV48), the keystone taxa of the diarrhea-phase module. During weaning, unclassified Muribaculaceae (ASV28 and ASV44), *UBA1819* (ASV151), *Barnesiella* (ASV497)*,* and *Ruminococcaceae*_*UCG-005* (ASV254) were identified being associated with non-diarrheic status, and they aggregated in the non-diarrhea module of co-occurrence patterns wherein unclassified Muribaculaceae (ASV28) and *Barnesiella* (ASV497) had a direct negative relationship with the members of the diarrhea module.

**Conclusions:**

Taken together, our results suggest that the dynamic successions of calf gut microbiota and the interactions among some bacteria could influence calf diarrhea, and some species of *Prevotella* might be the core microbiota in both neonatal and weaning calves, while species of Muribaculaceae might be the core microbiota in weaning calves for preventing calf diarrhea. Some ASVs affiliated with *Prevotella_2* (ASV4 and ASV26), *Prevotella_9* (ASV43), *Alloprevotella* (AVS14), unclassified Muribaculaceae (ASV28 and ASV44), *UBA1819* (ASV151), *Ruminococcaceae*_*UCG-005* (ASV254), and *Barnesiella* (ASV497) might be proper probiotics for preventing calf diarrhea whereas *Clostridium_sensu_stricto_1* (ASV48) might be the biomarker for diarrhea risk in specific commercial farms.

**Supplementary Information:**

The online version contains supplementary material available at 10.1186/s40104-022-00758-4.

## Background

Diarrhea is one of the most common causes of morbidity and mortality in young calves, especially dairy calves younger than one month [1, 2]. In the US, the National Animal Health Monitoring System estimated that more than half of the calf deaths were due to diarrhea and related diseases [3]. A similar mortality rate among dairy calves attributable to diarrhea was also reported in Korea [1]. Calf diarrhea not only incurs treatment cost but also lowers daily weight gain in calves [4], delays first conceptions [5], decreases milk production in the first lactation [6], consequently resulting in a great economic loss for dairy farms. Many pathogens have been implicated in calf diarrhea, including *Escherichia coli* K99^+^ (*E. coli* K99^+^), *Salmonella* spp*.*, *Clostridium perfringens*, *Cryptosporidium parvum*, bovine rotavirus (BRV), and bovine coronavirus (BCoV) [1]. Non-infectious factors such as improper colostrum management [7], weaning stress [8], and poor feeding environments can also increase the risk of calf diarrhea [9]. Moreover, oftentimes uncertain causality of diarrhea occurrence and cross infection together make diarrhea difficult to prevent and treat.

Diarrhea is much more common among young animals, especially after birth to shortly after weaning than among adult animals because of higher risk of enteric pathogens colonization in young animals than in adult animals [10, 11]. The high risk of colonization by pathogens in young animals stems from their underdeveloped gut and gut microbiota. Indeed, Kim et al. found that the absence of some species of Clostridia in the gut microbiota of neonatal mice made them unable to resist the colonization by *Salmonella enterica* Typhimurium or *Citrobacter rodentium* [12]. In addition, dysbiosis of gut microbiota induced by certain factors such as antibiotics and diseases can also lower resistance and increase enteric pathogen infection. Wu et al. demonstrated that dysbiosis of gut microbiota in rat induced by antibiotics lowered the resistance to colonization by *Salmonella* [13]. Compared to uninfected calves, calves infected with rotavirus have a higher abundance of *Escherichia*, *Clostridium_*g21, *Streptococcus*, and *Clostridium*, but a lower abundance of *Lactobacillus*, *Subdoligranulum*, *Blautia*, and *Coprococcus_*g2 in the fecal microbiota [14].

Weaning is a stressful process for young animals and can lower resistance to pathogenic colonization. Haag et al. found that infant mice after weaning were deficient in preventing their gut from colonization by *Campylobacter jejuni* [15]. During weaning, dairy calves are stressed nutritionally, and their gut microbiota undergoes compositional changes [16]. Typically, during weaning, the gut microbiota increases its diversity, and some important taxa, such as *Bacteroides*, *Blautia*, *Ruminococcus*, and *Succinivibrio*, may change their abundance [17]. The nutritional stresses coupled with changes in gut microbiota during and immediately after weaning increase the risk of enteric diseases, particularly diarrhea [17, 18].

Interestingly, some calves are prone to diarrhea, whereas others are resistant to diarrhea. In the 56-day experiment of Ma et. al [19], 13 out 42 milk replacer fed calves never exhibited diarrhea, 18 calves exhibited diarrhea and recovered after treated with electrolyte, but 11 calves exhibited diarrhea and needed to be treated with therapeutic antimicrobials. Ma et al. defined the latter two diarrhea status as resistant to diarrhea-induced dysbiosis, and susceptible to diarrhea-induced dysbiosis, respectively. The age of first diarrhea of these calves varied from d 8 to 19. Using the random forest machine learning algorithm with the microbiota collected from health calves at 7, 14, and 21 days of age and diarrhea calves prior to the onset of diarrhea, Ma et al. suggest that diarrhea could be predicted by the microbiota shift in early life. Kim et al. [20] described the similar age (5–50 days of age) fecal microbiota transplantation could ameliorate calf diarrhea with increasing the family Porphyromonadaceae. Therefore, we hypothesized that the gut microbiota might strongly correlate to calf diarrhea for some gut microbes might have resistance to the dysbiotic process with calf diarrhea from birth to post-weaning. To test our hypothesis, the stages with high incidence of diarrhea were identified in cohort from birth to shortly after weaning, and the fecal microbiotas between diarrheic calves and non-diarrheic calves in stages of high incidence of diarrhea, and between diarrhea phases (pre-diarrhea, diarrhea and post-diarrhea) were compared, and the diarrheic status- and diarrhea phase-associated amplicon sequence variants (ASVs) and modules were identified.

## Methods

### Animal experiment and sample collection

Two animal trials were conducted on a commercial farm located in Shaoxing (more than 3800 cows in stock), Zhejiang Province, China from September to November 2017 (trial 1) and November to December 2017 (trial 2). In trial 1, 14 female Holstein calves (initial bodyweight = 38.2 ± 2.0 kg, mean ± SD) were enrolled at birth. Immediately after birth, the calves were separated from their dams and moved to individual pens (1.0 m × 1.2 m) bedded with dry wheat straw and each offered 4 L of thawed colostrum (pooled the colostrum from other mothers before the trial, and stored at – 80 ºC) via an esophageal tube within 2 h after birthing. All neonatal calves in this trial were fed the same colostrum. From 1 to 5 days of age, each calf was bottle-fed 2 L of whole milk each at 0700, 1330, and 1800 h. During these 5 d, 4 g each of tylosin tartrate and sulfadimidine soluble (both powder) was mixed with the morning milk and fed to each calf for prophylaxis of pneumonia. At d 6, all the calves were moved to one group pen (8.0 m × 10.0 m) bedded with a rubber mat and dry wheat straw. The straw was replaced every other day. The calves had free access to a preset volume of whole milk (Additional file 1: Fig. S1) with an automated milk feeder system (Förster-Technik, Engen, Germany). Briefly, the calves get 6 L/d of whole milk from d 6 and this amount increased evenly up to 8 L/d on d 22. From d 23 to 41, each calf received 8 L of whole milk daily, and from d 42 to 62, the milk allowance was decreased evenly from 8 to 2 L/d. Milk supply was denied using the automated milk feeder system once a calf consumed 2 L of milk within 2 h during each meal to avoid overconsumption of milk. All the calves were weaned off milk at d 63. Calf starter pellets and a hay mixture of oat and alfalfa were offered ad libitum in the group pen. All the calves had free access to clean drinking water throughout the trial. Fecal samples were collected directly via rectal stimulation from every calf of the trial at d 1, 3, 5, 7, 9, 12, 15, 18, 28, 38, 48, 58, 62, 63, 65, 68, 73, and 78 (18 times in total). The fecal samples were placed in 2 mL nuclease-free tubes, immediately frozen in liquid nitrogen, then each month the 15 L liquid nitrogen tank with samples was transported to the lab and samples were stored at – 80 °C. The DNA of all the stored fecal samples was extracted in one month after the trial. When fecal samples were collected, fecal scores were recorded based on fecal fluidity [21]: 1 = normal, 2 = soft, 3 = runny, or 4 = watery. Calves that had a fecal score of 3 or 4 were considered diarrheic. No additional antibiotics were offered to calves after the diarrhea episode.

In trial 2, 43 female Holstein calves (initial bodyweight = 36.6 ± 1.9 kg, mean ± SD) were enrolled at birth. The calves were fed, nursed, and sampled exactly the same as for those in trial 1, but fecal samples were collected daily from d 8 to 18, which corresponded to the age when the first diarrhea peak was observed in trial 1.

### Metataxonomic analysis of the fecal microbiota

DNA was extracted from individual fecal samples according to the method of Zoetendal et al. with minimal modifications [22]. In brief, approximately 0.2 g of frozen fecal sample each was homogenized together with 1 mL of TE buffer using bead-beating (Biospec Products; Bartlesville, OK, United States) followed by phenol:chloroform-based DNA extraction. Agarose gel (1%) electrophoresis was performed to evaluate the DNA quality, and the DNA concentrations were determined using a NanoDrop 2000 spectrophotometer (Thermo Scientific, Waltham, MA, United States). The V3-V4 region of the 16S rRNA gene was amplified using primers 341F (5'-CCTAYGGGRBGCASCAG-3') and 806R (5'-GGACTACNNGGGTATCTAAT-3') to prepare amplicon libraries with each being labeled with a unique barcode sequence [23]. The amplicon libraries of all the samples were pooled in equal molar ratio and paired-end (2 × 250) sequenced on the Illumina HiSeq platform by Novogene Bioinformatics Technology Co., Ltd. (Tianjin, China). After demultiplexing, the paired-end sequencing reads were processed using the DADA2 package (version 1.8.0) in R and its pipeline [24]. Briefly, barcodes and primers were removed from the reads. Reads with more than 2 expected errors (maxN = 0, maxEE = c(2, 2), truncQ = 2) were filtered out. Dereplication and inference were performed using the DADA2 pipeline. After merging the paired reads and chimera filtering, an ASV table was constructed (to resolve bacteria at the species level [25]). The ASVs were taxonomically assigned based on the SILVA 16S rRNA gene database (version 132) [26] using a naive Bayesian classifier method [27] implemented in DADA2. The sequences of each sample were rarefied to same size with the minimum number of sequences in samples (35,137 sequences/sample in trial 1, and 19,986 sequences/sample in trial 2) using the ‘rarefy_even_depth' function in Phyloseq package (version 1.24.2) [28], and ASVs that appeared only once among all the samples were removed from the dataset. Alpha diversity metrics including observed species and Shannon diversity index were calculated using the Phyloseq package. Faith’s phylogenetic diversity (Faith’s PD) was calculated using the Picante package (version 1.8.2) in R, and evenness was calculated using the Microbiome package (version 1.12.0) in R. To minimize individual variance of calves, only the ASVs that were observed in at least 20% of calves at every single day (3 out of 14 calves in trial 1) or every single diarrheic status-associated phase (trial 2) were subjected to the downstream analysis.

### Pathogen detection

Major pathogens that can lead to calf diarrhea were tested in the fecal samples of trial 1 when diarrhea (fecal score ≥ 3) was first noted in a calf. *Salmonella* spp*.* was detected by PCR with specific primers (F:5′-TCGTCATTCCATTACCTACC-3′ and R:5′-AAACGTTGAAAAACTGAGGA-3′ [29] using fecal DNA samples. A commercial ELISA kit (BIO K 315, Bio-X Diagnostics, Rochefort, Belgique) each was used to detect BRV, BCoV, and *E. coli* (through its F5 attachment factor) in the fecal samples.

### Evaluation of the effects of age, diarrheic status, and diarrheic phases on fecal microbiota

A generalized linear mixed-effects model implemented in the nlme package (version 3.1.137) [30] in R was used to evaluate the effect of age and diarrhea on the alpha diversity metrics of the fecal microbiota, and Tukey’s all-pair comparison test using the ‘glht’ function in the multcomp package (version 1.4.8) [31] in R was used to do the multiple comparisons. In trial 1, the model included calf age and diarrheic status (diarrheic vs. non-diarrheic calves) among the calves as fixed effects and individual calves as random effect:

$$Y_{ijk} = \mu + A_{i} + H_{j} + I_{k} + \varepsilon_{ijk}$$

In trial 2, the model included diarrhea phases (pre-diarrhea, diarrhea, and post-diarrhea, see the results for the delineation of diarrhea phases) as fixed effect and individual calves as random effect:

$$Y_{mk} = \mu + S_{m} + I_{k} + \varepsilon_{mk}$$

where *Y*_*ijk*_ and Y_*mk*_ represent the variable of interest; *A*_*i*_ is the fixed effect of calf age; *H*_*j*_ is the fixed effect of calf diarrheic status, defined as non-diarrheic or diarrheic status based on fecal score; *S*_*m*_ is the fixed effect of calf diarrhea phases, defined as pre-diarrhea phase, diarrhea phase, or post-diarrhea phase based on the temporal changes of fecal scores of the same calves; *I*_*k*_ is the random effect of individual calves; and *ε*_*ijk*_ and *ε*_*mk*_ are the residual error. Non-parametric Kruskal–Wallis test was used to assess the effects of age and diarrhea on bacterial relative abundance, and Dunn’s all-pairs rank comparison test with *P* adjusted by false discovery rate was used to conduct multiple comparisons. A significant change was declared with *P* < 0.05.

### Fecal microbiota comparison among ages and identification of age-associated genera of bacteria

In trial 1, the overall fecal microbiotas between two ages were pairwise compared using analysis of similarity (ANOSIM) implemented in the Vegan package (version 2.5.3) [32] in R. When *P* < 0.05, the fecal microbiotas between two ages were considered completely different (*R*-value > 0.75), different (0.5 < *R*-value < 0.75), or tended to be different (0.3 < *R*-value < 0.5). *R*-value < 0.3 was considered not different.

Random forest regression was used to identify the fecal bacterial genera that were associated with the age of the calves using the randomForest package (version 4.6.14) [33, 34] in R. The genus table of all the samples was the input data. The random forest algorithm was executed with the default parameters (ntree = 1000, default mtry of p/3, where p is the number of input genera (‘features’)). The importance of a genus was ranked in the order of its ‘feature importance’, with feature importance being the decrease in prediction accuracy (in percent) of the model when that genus was removed. To explore the age-associated microbiota development, cross-validation was performed to estimate the optimal number of top-ranking age-associated genera required for prediction using the rfcv function implemented in the randomForest package in R. The identified genera were shown in a heatmap with their relative abundance. These genera were considered microbial markers of the respective ages.

### Fecal microbiota comparison between diarrheic statuses, between diarrhea phases, and identification of their associated ASVs and modules

Fecal microbiotas between the diarrheic and non-diarrheic calves in trial 1 and between diarrhea phases (pre-diarrhea, diarrhea, and post-diarrhea) in trial 2 were compared using principal coordinates analysis (PCoA) and permutational multivariate analysis of variance (PERMANOVA/adonis) based on Bray–Curtis dissimilarity. When the fecal microbiotas of diarrheic calves were significantly different from those of age-matched non-diarrheic calves, in trial 1, and when the fecal microbiotas of the calves with diarrhea differed significantly from those at their pre- or post-diarrhea phase in trial 2, Linear discriminant analysis Effect Size (LEfSe) [35] and significance test with DESeq2 [36] were used to identify the ASVs that might be associated with one of the diarrheic statuses or one of the diarrhea phases. Of the ASVs with an LDA score > 2 in LEfSe or an adjusted *P*-value < 0.05 in DESeq2, those with a log_2_ fold change > 1 (diarrheic/non-diarrheic calves, or diarrhea/pre- or post-diarrhea phase) were considered to be associated with diarrheic status or phase, whereas those with a log_2_ fold change < – 1 (defined the same as above) were considered associated with non-diarrheic status or phase.

Co-occurrence patterns of the fecal microbiotas of the diarrheic and non-diarrheic calves (see the results for the delineation of the stages) in trial 1, or the fecal microbiota of the calves at different diarrhea phases were examined using the SparCC algorithm [37] with ASV count table as the input data. The pattern was visualized using the igraph package (version 1.2.5) [38] in R, and correlations with a *P* < 0.05 and a co-efficient R ≥ 0.5 or ≤ – 0.5 being considered positive and negative correlations, respectively. Modules of the co-occurrence patterns were generated using the walktrap algorithm [39] implemented in igraph. Modules with less than 3 nodes were deleted from the co-occurrence patterns. The identified ASVs associated with a diarrheic status or diarrhea phase were highlighted in the patterns. The modules aggregated with ASVs that were associated with diarrhea in trial 1 or with the diarrhea phase in trial 2 were considered as diarrhea modules and diarrhea-phase modules, respectively, whereas the modules formed with ASVs that were not with diarrhea in trial 1 or associated with pre- or post-diarrhea phases in trial 2 were considered as non-diarrhea modules or pre- or post-diarrhea modules.

## Results

### Trial 1

#### Development of fecal bacterial microbiota of calves and temporal microbial successions

**Fig. 1:**
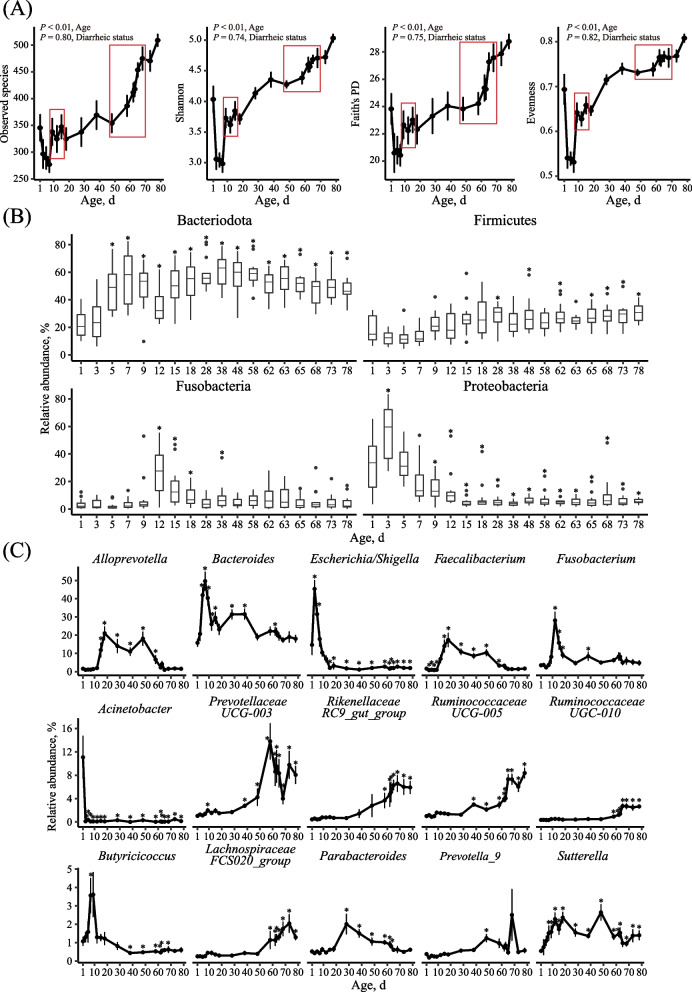
Dynamic changes of fecal bacterial microbiota of calves from birth to post-weaning (trial 1). Dynamic changes of alpha diversity metrics with the red boxes indicating the diarrhea peaks (**A**), bacterial phyla (**B**), and major genera (**C**) across ages. Only the phyla and genera each with a relative abundance > 1% in at least 60% of samples at any single age were shown. The relative abundance significantly differing from that of d 1 is indicated with a * (*P* < 0.05)

In total, 15,283,464 quality-filtered amplicon sequences were obtained from 251 fecal samples (the fecal sample of calf Y03 on d 5 was not analyzed due to contamination with wheat straw) with an average of 60,890 ± 7193 (mean ± SD) sequences per sample. The sequencing depth coverage reached > 99.96% on average (99.89% to 100.00%). In the fecal samples collected from 1 day of age, 345 ASVs (referred to as species hereafter) on average per sample were identified with a Shannon diversity index of 4.03 (Fig. 1A). The number of observed species, Faith’s PD, Shannon diversity index, and evenness decreased from d 1 to 7 but recovered at d 9. Then, observed species, Faith’s PD, Shannon index, and evenness gradually increased, though with fluctuation, to about 509, 28.77, 5.03 and 0.81, respectively, at d 78 (Fig. 1A). Over this period, age significantly (*P* < 0.05) affected all the diversity metrics, whereas diarrheic status did not affect (*P* > 0.05) any of these four metrics.

Collectively, the ASVs were classified into 200 genera within 12 phyla. Bacteroidota, Firmicutes, Proteobacteria, and Fusobacteria each had a relative abundance > 1% in more than 60% of the fecal samples at any day (Fig. 1B). Of the 200 bacterial genera identified across all the fecal samples, 15 genera each had a relative abundance  > 1% in at least 60% of the fecal samples at a single day. These genera included *Alloprevotella*, *Bacteroides*, *Escherichia/Shigella*, *Faecalibacterium*, *Fusobacterium*, *Acinetobacter*, *Prevotellaceae*_*UCG-003*, *Rikenellaceae*_*RC9_gut_group*, *Ruminococcaceae*_*UCG-005*, *Ruminococcaceae*_*UGC-010*, *Butyricicoccus*, *Lachnospiraceae*_*FCS020_group*, *Parabacteroides*, *Prevotella*_*9* and *Sutterella.* These predominant genera displayed temporal changes in relative abundance over the course of the trial (Fig. 1C). *Alloprevotella*, *Faecalibacterium*, *Parabacteroides*, and *Sutterella* increased their relative abundance (*P* < 0.05) and maintained a higher abundance around d 15 to 58 compared to d 1 and then decreasing towards the end of the trial. Compared to d 1, *Bacteroides*, *Escherichia/Shigella*, *Fusobacterium*, and *Butyricicoccus* increased and then decreased their relative abundance sharply (*P* < 0.05) at around d 10*.* On the contrary, *Prevotellaceae*_*UCG-003*, *Rikenellaceae*_*RC9_gut_group*, *Ruminococcaceae*_*UCG-005*, *Ruminococcaceae*_*UGC-010*, and *Lachnospiraceae*_*FCS020_group* increased their relative abundance (*P* < 0.05) during the later days of the trial and maintained or decreased their relative abundance until the end of the trial. *Acinetobacter* differed from all the other genera as it lost its initial high relative abundance dramatically by d 3 (*P* < 0.05) and never recovered. *Prevotella*_*9* only increased (*P* < 0.05) on d 9.

#### Composition and distribution of age-associated bacterial genera from birth to post-weaning

Pair-wise comparison of the fecal microbiotas at the ASV level among the 18 time points using ANOSIM revealed three age periods, with the first, second, and third age periods being from d 1 to 12, 15 to 63, and 58 to 78, respectively. The fecal microbiotas were similar (*R*-value < 0.5) within each age period but different between most of the two age periods (Table 1).

**Table 1:** A matrix of *R*-values of pair-wise comparison of the fecal microbiota of trial one at the ASV level using ANOSIM

*R*	1	3	5	7	9	12	15	18	28	38	48	58	62	63	65	68	73	78
1	0																	
3	0.43^*^	0																
5	0.41^*^	0.20^*^	0															
7	0.45^*^	0.40^*^	0.10^*^	0														
9	0.43^*^	0.62^*^	0.38^*^	**0.07**	0													
12	0.46^*^	0.73^*^	0.60^*^	0.41^*^	0.25^*^	0												
15	0.57^*^	0.89^*^	0.83^*^	0.70^*^	0.53^*^	**0.09**	0											
18	0.68^*^	0.92^*^	0.91^*^	0.83^*^	0.74^*^	0.39^*^	**0.02**	0										
28	0.68^*^	0.97^*^	0.96^*^	0.86^*^	0.67^*^	0.59^*^	0.24^*^	0.20^*^	0									
38	0.67^*^	0.96^*^	0.96^*^	0.83^*^	0.64^*^	0.55^*^	0.37^*^	0.44^*^	0.12^*^	0								
48	0.66^*^	0.95^*^	0.93^*^	0.87^*^	0.73^*^	0.59^*^	0.28^*^	0.18^*^	0.10^*^	0.11^*^	0							
58	0.64^*^	0.91^*^	0.89^*^	0.76^*^	0.63^*^	0.56^*^	0.49^*^	0.58^*^	0.48^*^	0.29^*^	0.27^*^	0						
62	0.61^*^	0.88^*^	0.84^*^	0.78^*^	0.60^*^	0.48^*^	0.45^*^	0.60^*^	0.51^*^	0.30^*^	0.30^*^	**0.03**	0					
63	0.62^*^	0.90^*^	0.86^*^	0.76^*^	0.58^*^	0.47^*^	0.42^*^	0.56^*^	0.53^*^	0.34^*^	0.31^*^	**0.00**	**-0.06**	0				
65	0.68^*^	0.88^*^	0.83^*^	0.77^*^	0.56^*^	0.57^*^	0.57^*^	0.75^*^	0.73^*^	0.57^*^	0.63^*^	0.27^*^	**0.08**	**0.04**	0			
68	0.61^*^	0.83^*^	0.81^*^	0.76^*^	0.59^*^	0.51^*^	0.52^*^	0.68^*^	0.68^*^	0.57^*^	0.60^*^	0.42^*^	0.24^*^	0.19^*^	**0.01**	0		
73	0.63^*^	0.90^*^	0.86^*^	0.79^*^	0.60^*^	0.57^*^	0.56^*^	0.76^*^	0.74^*^	0.59^*^	0.64^*^	0.39^*^	0.20^*^	0.15^*^	**0.01**	**-0.03**	0	
78	0.72^*^	0.95^*^	0.94^*^	0.87^*^	0.71^*^	0.65^*^	0.67^*^	0.85^*^	0.87^*^	0.77^*^	0.79^*^	0.56^*^	0.36^*^	0.28^*^	0.14^*^	**0.07**	**-0.05**	0

^*^ Represents the pair-wise comparison with *P*-value < 0.05. Those with *R*-value < 0.5 are blod

With *P*-value < 0.05, the fecal microbiotas of two ages were considered completely different at *R*-value > 0.75; different at 0.5 < *R*-value < 0.75; tended to be different at a 0.3 < *R*-value < 0.5; not different at *R*-value < 0.3

**Fig. 2:**
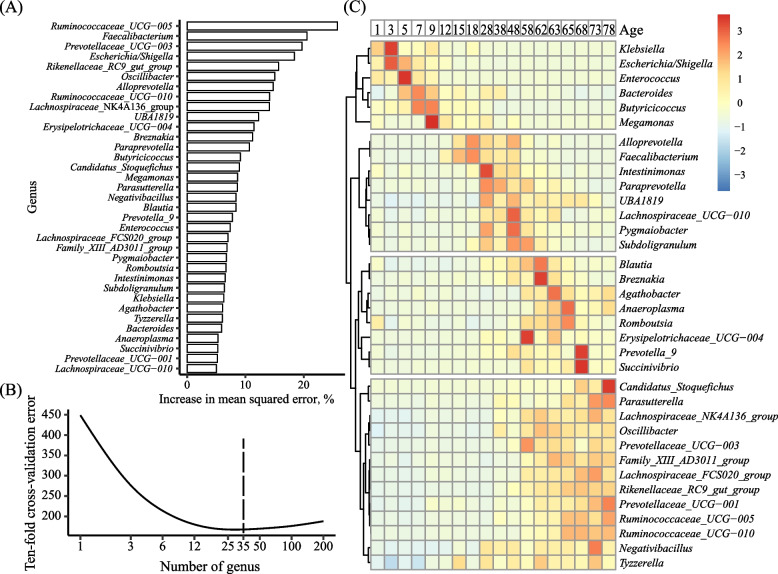
Age-associated bacterial genera from birth to post-weaning (trial 1). The top 35 age-associated bacterial genera ranked by importance to the accuracy of the random forest regression model (**A**). Ten-fold cross-validation error as a function of the number of input genera was used to regress against the chronologic age of calves. The dotted line indicates the 35 genera used in the model (**B**). Heatmap of the top 35 age-associated genera and the clusters they formed based on their relative abundance across ages (**C**)

Thirty-five age-associated bacterial genera were identified by random forest regression (Fig. 2A and B), and they were distributed in 4 clusters (Fig. 2C) each corresponding to one of the age periods (Table 1). Of these age-associated genera, *Klebsiella*, *Escherichia/Shigella*, *Enterococcus*, *Bacteroides*, *Butyricicoccus* and *Megamonas* in the first cluster were predominant at the early age (d 1 to 12); *Alloprevotella*, *Faecalibacterium*, *Intestinimonas*, *Paraprevotella*, *UBA1819*, *Lachnospiraceae_UCG-010*, *Pygmaiobacter*, and *Subdoligranulum* in the second cluster were predominant at d 15 to 58; *Blautia*, *Breznakia*, *Agathobacter*, *Anaeroplasma*, *Romboutsia*, *Erysipelotrichaceae*_*UCG-004*, *Prevotella*_*9*, and *Succinivibrio* in the third cluster and *Candidatus_Stoquefichus*, *Parasutterella*, *Lachnospiraceae*_*NK4A136_group*, *Oscillibacter*, *Prevotellaceae*_*UCG-003*, *Family_XIII_AD3011_group*, *Lachnospiraceae*_*FCS020_group*, *Rikenellaceae*_*RC9_gut_group*, *Prevotellaceae*_*UCG-001*, *Ruminococcaceae*_*UCG-005*, *Ruminococcaceae*_*UCG-010*, *Negativibacillus*, and *Tyzzerella* in the fourth cluster were predominant at d 48 to 68 and d 58 to 78, respectively.

#### Diarrhea characteristics of the study cohort

**Fig. 3:**
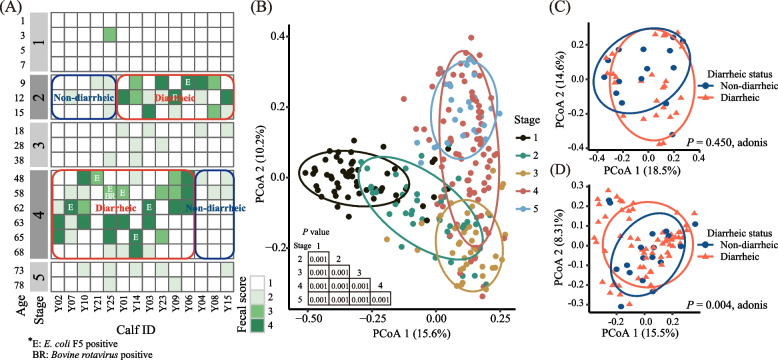
Development stages of the fecal microbiota based on fecal score and the comparison of the overall fecal microbiota from birth to post-weaning (trial 1). Development stages of the 14 calves (**A**). Principal coordinates analysis (PCoA) plots comparing the fecal microbiotas among the 5 stages (**B**) and between diarrheic and non-diarrheic calves at stage 2 (**C**) and stage 4 (**D**). Age refers to days after birth. The gradual weaning started at d 42 and ended at d 63

Over the course of the trial 1, all the calves had fecal score ≥ 3 at least one sampling day (Fig. 3A). Based on the fecal scores of all the calves, the 78 d of trial was divided into five stages: stage 1: d 1 to 7, before the first diarrhea peak; stage 2: d 9 to 15, the first diarrhea peak; stage 3: d 18 to 38, the stage after the first peak but before the second diarrhea peak; stage 4: d 48 to 68, the second diarrhea peak; and stage 5: d 75 to 78, the stage after the second diarrhea peak. All the 14 calves were non-diarrheic in stages 1, 3, and 5. Pathogen detection showed that all the 20 diarrheic fecal samples (9 in the first and 11 in the second diarrhea peaks) were *Salmonella* spp. and BCoV negative, but eight were *E. coli* K99^+^ positive (2 in the first and 6 in the second diarrhea peaks). One of the diarrheic samples (in the second diarrhea peak) was both BRV and *E. coli* K99^+^ positive (Fig. 3A).

#### ASVs and modules associated with diarrheic status

The fecal microbiotas differed among the five stages (*P* < 0.001, Fig. 3B). The fecal microbiotas of diarrheic and non-diarrheic calves did not differ (*P* = 0.450) in stage 2 (Fig. 3C) but did differ (*P* = 0.004) in stage 4 (Fig. 3D). Based on fold change and LEfSe or DESeq2 analysis, 147 diarrheic status-associated ASVs were identified in stage 4 including 91 diarrhea-associated ASVs and 56 non-diarrhea-associated ASVs (Additional file 2: Fig. S2). The diarrhea-associated ASVs mainly consisted of *Bacteroides* (15 ASVs), *Ruminococcaceae*_*UCG-005* (8 ASVs), *Ruminococcaceae*_*UCG-010* (6 ASVs), *Ruminococcaceae*_*UCG-013* (4 ASVs) and *Lachnospiraceae*_*FCS020_group* (4 ASVs). The non-diarrhea-associated ASVs mainly consisted of *Ruminococcaceae*_*UCG-005* (8 ASVs), *Flavonifractor* (4 ASVs) and *Bacteroides* (3 ASVs).

**Fig. 4:**
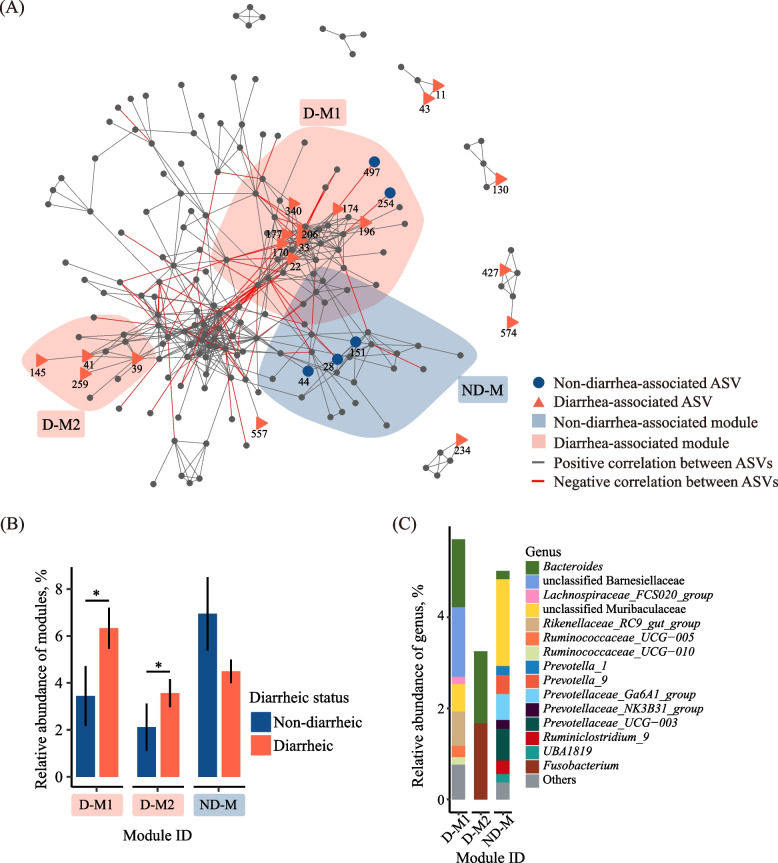
Co-occurrent pattern of the fecal microbiota at stage 4 in trial 1. Pattern showing the diarrhea status-associated ASVs and modules (**A**). Mean relative abundance of diarrhea status-associated modules in diarrheic and non-diarrheic calves (**B**). ASVs relative abundance at genus level in different modules (**C**). ASV254 and ASV497 in module 2 were not included in plots (**B**) or (**C**)

The co-occurrence pattern for stage 4 had 195 nodes, 603 edges, and 17 modules (Fig. 4A, Additional file 3: Table S1). Nineteen diarrhea-associated ASVs and 5 non-diarrhea-associated ASVs showed up in the co-occurrence pattern. The diarrhea-associated ASVs including *Prevotellaceae_UCG-003* (ASV11, ASV43), *Ruminococcaceae_UCG-010* (ASV427, ASV574), *Butyricicoccus* (ASV130), *Lachnospiraceae_FCS020_group* (ASV234) and unclassified Ruminococcaceae (ASV557) were scattered without aggregation in any of the modules. *Bacteroides* (ASV170, ASV206), *Rikenellaceae_RC9_gut_group* (ASV33), *Ruminococcaceae_UCG-010* (ASV196), *Lachnospiraceae_FCS020_group* (ASV174), unclassified Bacteroidales (ASV177), unclassified Barnesiellaceae (ASV22) and unclassified Ruminococcaceae (ASV340) were aggregated in diarrhea module 1 (D-M1), while *Bacteroides* (ASV39, ASV41, ASV145, and ASV259) were aggregated in diarrhea module 2 (D-M2). The non-diarrhea-associated Muribaculaceae (ASV28 and ASV44) and *UBA1819* (ASV151) were aggregated in non-diarrhea module (ND-M). The non-diarrhea-associated *Barnesiella* (ASV497) and *Ruminococcaceae*_*UCG-005* (ASV254) were aggregated in D-M1, and they formed negative correlations with the ASVs in D-M1. The ASVs in D-M1 and D-M2 had a higher (*P* < 0.05) total relative abundance in diarrheic calves than in non-diarrheic calves (Fig. 4B). The D-M1 was mainly occupied by *Bacteroides*, unclassified Barnesiellaceae*,* and *Rikenellaceae_RC9_gut_group*, with 1.49%, 1.53% and 0.75% relative abundance, respectively; D-M2 was occupied by *Fusobacterium* and *Bacteroides*, with 1.67% and 1.58% relative abundance, respectively, but the ND-M was mainly occupied by unclassified Muribaculaceae, *Prevotellaceae_Ga6A1_group*, *Prevotellaceae_UCG-003*, and *Prevotella_9*, with 1.90%, 0.69%, 0.57%, and 0.42% relative abundance, respectively (Fig. 4C).

### Trial 2

#### Diarrhea characteristics of the study cohort

**Fig. 5:**
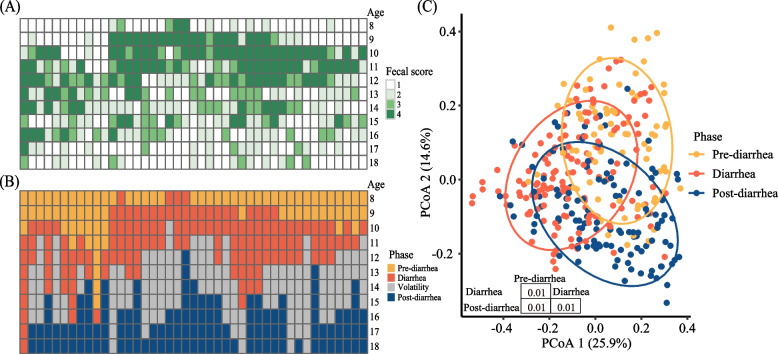
Transition phase division of the 43 calves based on fecal score in trial 2. Fecal score of the 43 calves from d 8 to 18 (**A**). Phase division based on samples fecal score (**B**). Principal coordinates analysis (PCoA) plot of fecal microbiotas among pre-diarrhea, diarrhea, and post-diarrhea phases (**C**)

Over the course of the trial 2, all the calves had fecal score ≥ 3 at least 1 d. (Fig. 5A). In the 43 calves, 4, 20, 33, 31, 30, 18, 18, 16, 15, 4 and 5 calves had diarrhea on d 8 to 18 respectively. In which, 32 calves had one or more episodes of diarrhea 1 to 5 d after recovering from a previous episode (Fig. 5A). Except of d 9, 10 and 11, the microbial composition had no significant difference between diarrheic and non-diarrheic calves within the same age (Additional file 4: Fig. S3). At the same age, calves were in different days of diarrhea, so microbial changes were also out of synchronization (Fig. S3). So based on the temporal changes of the fecal scores, the fecal samples were divided into four phases: pre-diarrhea (when fecal score  < 3), diarrhea (fecal scores ≥ 3 consecutively), post-diarrhea (fecal score falling below 3), and volatility (fecal score rising to ≥ 3 after 1 to 5 d below 3) (Fig. 5B). To track the microbial changes from the pre-diarrhea to post-diarrhea, samples of calves of different ages with or without diarrhea were combined into phases for analysis.

#### Changes in fecal bacterial communities of calves from pre- to post-diarrhea

In total, 18,706,378 quality-filtered amplicon sequences were obtained from 340 fecal samples (the fecal samples of pre-diarrhea, diarrhea, and post-diarrhea phases) with an average of 55,019 ± 10,329 (mean ± SD) sequences per sample. The sequencing depth coverage reached > 99.97% on average (99.91% to 99.99%). The fecal microbiotas differed among pre-diarrhea, diarrhea, and post-diarrhea phases (*P* < 0.01, Fig. 5C).

**Fig. 6:**
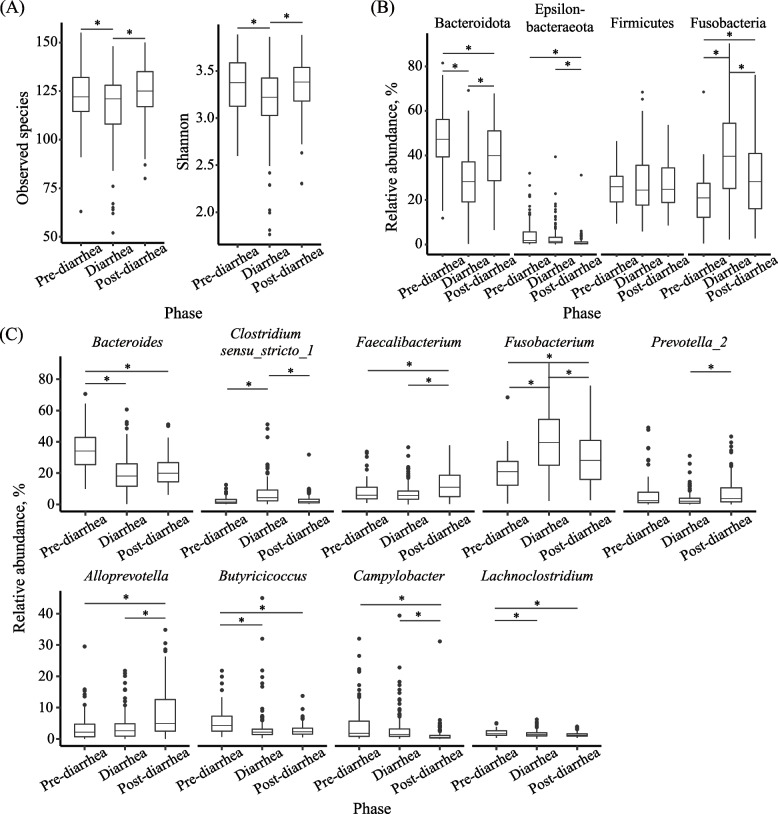
Fecal microbiota changes from pre-diarrhea, diarrhea, and post-diarrhea phases in trial 2. Two alpha diversity metrics of the fecal microbiota (**A**), Relative abundance of predominant bacterial phyla (**B**) and genera (**C**). Only the phyla and genera each with a relative abundance > 1% in at least 60% of samples in a single phase were shown. Significant differences between two phases were indicated with a * (*P* < 0.05)

The lowest number of observed species (on average 117 ASVs per sample) and Shannon index (3.2) were found in the fecal samples of the diarrhea phase (Fig. 6A). Both metrics increased (*P* < 0.05) in the post-diarrhea phase. Of the major phyla and genera (each with a relative abundance > 1% in > 60% of the fecal samples at any phase), the phylum Bacteroidota decreased (*P* < 0.05), while the phylum Fusobacteria increased (*P* < 0.05) from the pre-diarrhea to diarrhea phase (Fig. 6B), and the genera *Bacteroides*, *Butyricicoccus*, and *Lachnoclostridium* decreased (*P* < 0.05), while *Clostridium_sensu_stricto_1* and *Fusobacterium* increased (*P* < 0.05) during the phase transition (Fig. 6C). In the post-diarrhea phase, the phyla Bacteroidota and Fusobacteria and the genus *Fusobacterium* tended to return their relative abundance to the pre-diarrhea level (*P* < 0.05), while the genus *Clostridium_sensu_stricto_1* lost the relative abundance gained in the diarrhea phase (*P* > 0.05), and the genera *Bacteroides*, *Butyricicoccus*, and *Lachnoclostridium* maintained their relative abundance of the diarrhea phase. Although not having changed their relative abundance from the pre-diarrhea phase to the diarrhea phase, the genera *Faecalibacterium*, *Prevotella_2*, and *Alloprevotella* increased their relative abundance (*P* < 0.05), while the phylum Epsilonbacteraeota and the genus *Campylobacter* decreased their relative abundance (*P* < 0.05) in the post-diarrhea phase.

#### ASVs and modules associated with the diarrhea phase

**Fig. 7:**
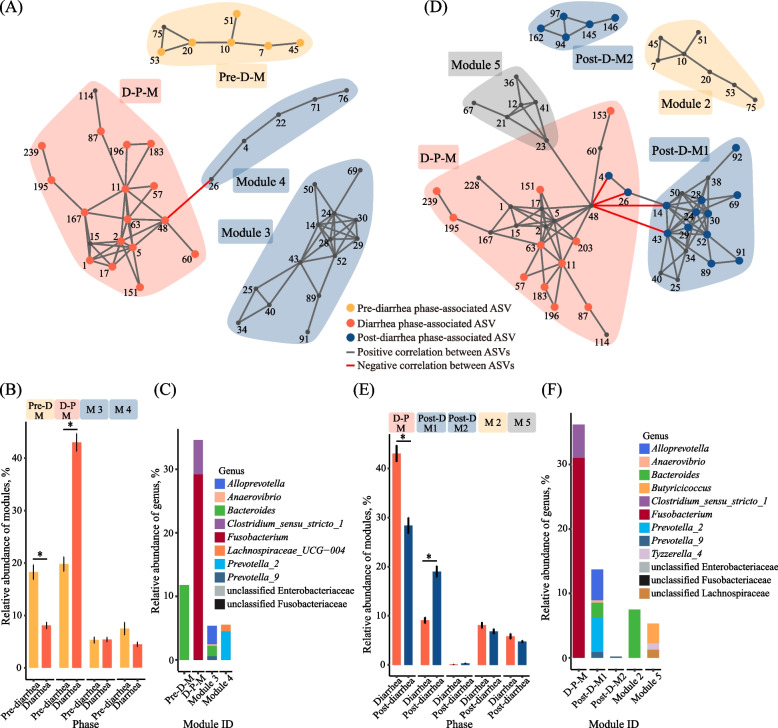
Co-occurrent pattern of the fecal microbiota among three phases in trial 2. Pattern among pre-diarrhea and diarrhea phases (**A**). Mean relative abundance of phase-associated modules in pre-diarrhea and diarrhea (**B**). ASVs relative abundance at genus level in pre-diarrhea and diarrhea modules (**C**). Pattern among diarrhea and post-diarrhea phases (**D**). Mean relative abundance of phase-associated modules in diarrhea and post-diarrhea (**E**). ASVs relative abundance at genus level in diarrhea and post-diarrhea modules (**F**). ASV4 and ASV26 in diarrhea-M were divided into post-D-M1 in plot (**E**) and (**F**)

Comparison of the fecal microbiotas between pre-diarrhea and diarrhea phases revealed (based on fold change and analysis using LEfSe or DESeq2) 32 pre-diarrhea-associated ASVs and 29 diarrhea phase-associated ASVs (Additional file 5: Fig. S4). The pre-diarrhea phase-associated ASVs mainly consisted of *Bacteroides* (12 ASVs), *Parabacteroides* (4 ASVs), *Butyricicoccus* (3 ASVs), and *Flavonifractor* (3 ASVs). The diarrhea phase-associated ASVs mainly consisted of *Clostridium_sensu_stricto_1* (8 ASVs), unclassified Fusobacteriaceae (7 ASVs), *Fusobacterium* (6 ASVs), and unclassified Enterobacteriaceae (3 ASVs). The co-occurrence pattern of the pre-diarrhea and diarrhea phases had 44 nodes, 78 edges, and 4 modules (Fig. 7A). The pre-diarrhea and diarrhea phase-associated modules (pre-D-M and D-P-M, respectively) were aggregated with six and 16 pre-diarrhea and diarrhea phase-associated ASVs, respectively, and both modules were independent. Module 3 and module 4 were aggregated with 14 and 5 ASVs, respectively. Both module 3 and module 4 had no diarrhea or pre-diarrhea phase-associated ASVs, but module 4 formed a negative correlation with the D-P-M through *Prevotella_2* (ASV26) and *Clostridium_sensu_stricto_1* (ASV48). The ASVs in the D-P-M had a higher (*P* < 0.05) total relative abundance in the diarrhea phase than in the pre-diarrhea phase, whereas the opposite was true for the ASVs in pre-D-M (Fig. 7B). The D-P-M was occupied by ASVs assigned to *Clostridium_sensu_stricto_1* and *Fusobacterium*, with a relative abundance of 29.11% and 5.42%, respectively. The pre-D-M was only occupied by ASVs of *Bacteroides*, with a relative abundance about 11.77%. The ASVs of module 3 mainly consisted of *Allprevotella*, *Bacteroides* and *Prevotella*_*9*, with 2.94%, 1.65% and 0.55% relative abundance, respectively. The ASVs of module 4 were classified to *Prevotella_2* and *Lachnospiraceae_UCG-004*, with 4.48% and 1.05% relative abundance, respectively (Fig. 7C).

When compared the ASVs between diarrhea and post-diarrhea phases, 44 post-diarrhea phase-associated ASVs and 23 diarrhea phase-associated ASVs were identified based on fold change and analysis using LEfSe or DESeq2 (Additional file 4: Fig. S4). The post-diarrhea phase-associated ASVs mainly consisted of *Prevotella_9* (13 ASVs), *Alloprevotella* (8 ASVs), *Bacteroides* (4 ASVs), and *Collinsella* (3 ASVs). The diarrhea phase-associated ASVs mainly consisted of *Clostridium_sensu_stricto_1* (6 ASVs), unclassified Fusobacteriaceae (7 ASVs), and unclassified Enterobacteriaceae (4 ASVs). The co-occurrence pattern constructed of the fecal microbiotas at post-diarrhea and diarrhea phases had 57 nodes, 116 edges, and 5 modules (Fig. 7D). The D-P-M was aggregated with 11 diarrhea phase-associated ASVs and two post-diarrhea phase-associated ASVs (ASV4 and ASV26, both belonging to *Prevotella*_2), both of which had a negative relationship with *Clostridium_sensu_stricto_1* (ASV48), a diarrhea phase-associated ASV and the keystone of D-P-M. Two post-diarrhea phase-associated modules (post-D-M1 and post-D-M2) were identified in the co-occurrence network, and post-D-M1 and post-D-M2 were aggregated with 11 and 5 post-diarrhea phase-associated ASVs, respectively. The composition of post-D-M1 was similar to that of module 3 in Fig. 7A, but post-D-M1 had more betweenness and close centrality than module 3 and formed a negative correlation with D-P-M through *Alloprevotella* (ASV14) and *Prevotella_9* (ASV43), both of which negatively associated with *Clostridium_sensu_stricto_1* (ASV48) in the D-P-M. The ASV composition of module 2 was the same as that of pre-D-M in Fig. 7A, and module 2 was standalone from the other four modules (Fig. 7D). Module 5 consisted of six ASVs. Although having no diarrhea and post-diarrhea phase-associated ASVs, module 5 positively connected with D-P-M through *Tyzzerella_4* (ASV23) and *Clostridium_sensu_stricto_1* (ASV48). The D-P-M was occupied by *Fusobacterium*, *Prevotella*_*2* and *Clostridium_sensu_stricto_1* with a relative abundance of 30.94%, 5.34% and 5.19%, respectively (Fig. 7F). The post-D-M1 was mainly occupied by *Alloprevotella, Bacteroides* and *Prevotella_9*, with a combined relative abundance of 4.80%, 2.32%, and 0.88%, respectively. The post-D-M2 was only occupied by *Prevotella_9*, with a relative abundance of 0.21%.

## Discussion

A better understanding of the fecal microbiota in diarrheic and non-diarrheic calves can inform improved treatment and prevention strategies. Fecal microbiotas between diarrheic and non-diarrheic calves have been compared after interventions, such as trehalose supplementation [40], feeding waste milk containing antibiotic residues [41] or supplemented with sodium humate and glutamine combination [42], or single species [43] or multispecies probiotics [44]. These interventions affected the developing process of gut microbiota and increased the abundance of *Bifidobacterium* and *Lactobacillus*, which might conceal the natural development of resistance to pathogenic colonization in pre-weaned calves. Without these types of interventions, Kim et al. [20] described the ability of a fecal microbiota transplantation (inclusion was collected from the health calves of the similar age to diarrheic calves) could ameliorate diarrhea and restore gut microbial composition in pre-weaning calves. Most of these comparative studies focused on pre-weaning calves. In the present study, we first examined the dynamic development of the gut microbiota (represented by fecal microbiota) and diarrhea occurrence in dairy calves from birth to 15 d post-weaning with frequently fecal sampling in trial 1, which allowed us to identify two diarrhea peaks. Then we analyzed the fecal microbiota and diarrhea occurrence of another group of dairy calves over 11 d corresponding to the first diarrhea peak with fecal samples collected daily. Trial 1 helped test our hypothesis that some gut microbes might have resistance to dysbiotic process with calf diarrhea by dictating the microbial co-occurrence patterns during weaning, while trial 2 allowed us to examine in details as the calves transitioned from pre-diarrhea to diarrhea and then to post-diarrhea phases shortly after birth.

The development of the gut microbiota appeared to have three age periods with birth and weaning as the separatrices [45, 46]. As antibiotics had been used from d 1 to 5, the drastically decreasing of bacterial richness after birth (Fig. 1A) might be the effects of tylosin tartrate and sulfadimidine. However, in the recent study of neonatal dairy calves, without antibiotics, a similar decrease of bacterial richness was reported by Kim et al. [47] and Klein-Jöbstl et al. [48]. In the first two weeks after birth, the underdeveloped gut was colonized primarily by facultatively anaerobic microbes, especially *Escherichia/Shigella*, to render the intestinal environment suitable for anaerobic intestinal microbes to colonize, so the decrease of bacterial richness might be a result of bacterial adaptation. *Escherichia/Shigella* had a low relative abundance in the fecal samples at any day after birth to 15 d after weaning, but its relative abundance increased up to 45.36% and 31.51% on d 3 and 5, respectively, and then decreased to around 2% since d 15. The increased colonization by *Escherichia/Shigella* in young calves has been described previously [49], and it might explain the susceptibility of calves to diarrhea caused by *E. coli*. *Klebsiella*, which belongs to the same family, Enterobacteriaceae*,* as *Escherichia*/*Shigella*, also appeared to be an age-associated bacterial genus for this period. *Klebsiella pneumoniae* [50] and *Klebsiella oxytoca* [51] are opportunistic pathogenic in humans and are associated with increased infection mortality rate, particularly in immunocompromised individuals, neonates, and the elderly. However, infections in calves caused by *Klebsiella* have not been reported frequently. Glantz and Jacks reported that *Klebsiella* spp. occurred naturally in calves, and they might be responsible for some mortality [52]. Komatsu et al. reported fatal suppurative meningoencephalitis caused by *K. pneumoniae* in two calves [53]. Aslan et al. reported that *K. pneumoniae* could be isolated in calves suffering from respiratory tract infection, which was not cured by florfenicol [54]. In the present study, we observed changes of relative abundance of *Klebsiella*, decreasing from 1.07% and 2.09% on d 1 and 3, respectively, to less than 0.1% at d 5. Tylosin tartrate and sulfadimine are both broad spectrum antibiotics exerting their antimicrobial action by inhibiting the bacterial protein synthesis [55], and competing with para-aminobenzoic acid for dihydropteroate synthase [56], respectively. The decrease in *Klebsiella* might be a result of tylosin tartrate [57]. The peak of *Klebsiella* abundance did not correspond to a diarrhea peak, but it should be given special attention in calf industry because of its pathogenic significance.

*Bacteroides* was the most abundant genus through the whole period, but its relative abundance increased sharply to 41.96%, 49.66%, and 40.38% at d 5, 7, and 9, respectively, so it could be an age-associated bacterial genus for the first two weeks after birth. Its high abundance was attributed to its stronger saccharolytic ability than *Prevotella* in the gut of young calves [58, 59]. The acetate produced by *Bacteroides* could be consumed by other bacteria, such as *Butyricicoccus* and *Megamonas*, to produce butyrate and propionate [60], both of which are the main source of energy for intestinal epithelial cells, and butyrate can also inhibit the signaling pathways of pro-inflammatory cytokines [61], enhance intestinal barrier function by increasing mucin secretion and enhancing the tight-junctions [62]. Thus, the colonization by these microbes in the gut of young calves might facilitate the establishment of a functional gut. From the third week onward, weaning separated the microbiota characteristic of the two periods. Prior to weaning for more than 40 d, the microbiotas remained similar, suggesting that the gut might have established a stable functional microbiota over that period, with limited recruitment. However, with the transition from liquid to total solid feed, some of the gut bacteria displayed considerable changes, as exemplified by the replacement of *Alloprevotella*, *Faecalibacterium*, and *Parabacteroides* by *Prevotellaceae_UCG-003*, *Rikenellaceae_RC9_gut_group*, *Ruminococcaceae_UCG-005*, *Ruminococcaceae_UGC-010*, and *Lachnospiraceae_FCS020_group*. These five genera are within families that contain species capable of utilizing structural polysaccharides, and this replacement might facilitate the degradation of structural polysaccharides and the stability of the gut microbiota. Future research can help identify the species of these five genera and characterize their functions. Two diarrhea peaks were observed during the three developing periods of the gut microbiota, and less than half of the tested diarrheic fecal samples were pathogen positive, which suggests that microbiota homeostasis may be more important in preventing diarrhea than directly killing pathogens. Comparing fecal microbiota transplantation and antibiotic treatment for ameliorating calf diarrhea, Kim et al. [20] confirmed that gut microbial manipulation could offer another therapeutic paradigm, beyond antibiotic based therapies. Our data also suggest that prophylaxis/preventions with probiotics should be better administered in d 1 to 7 (stage 1, before the first diarrhea peak) and d 18 to 38 (stage 3, the stage after the first peak but before the second diarrhea peak). Supplementation of newborn calves with *Lactobacillus* and *Bifidobacterium* [63] or *Faecalibacterium prausnitzii* [64] within the first 7 d of life decreased diarrhea, but no study was found in the literature that had tested probiotic supplementation in stage 3. To promote the natural development of the gut microbiotas during the transition, the potential probiotics in the gut of calves should be identified.

Comparison of the fecal microbiota using LEfSe and the subsequent identification of the correlations between the differential bacterial genera and the core bacterial genera in the gut is a well-trodden path to reveal the effects of diarrhea on the microbiota and find potential probiotics in neonatal dairy calves for preventing diarrhea [65]. But many standard correlation analyses may lead to misleading results because 16S rRNA gene profiling data are sparse and compositional [37]. SparCC, which is tailored to the compositional and sparse features of genomic survey data and allows for inference of correlations between genes or species, has been used to elucidate the networks of interaction among microbial species living in or on the human body [37, 66]. Therefore, to reduce the incidence of false positive results, three levels of evaluation criteria including co-occurrence patterns examined using the SparCC algorithm (correlations with a *P* < 0.05 and a co-efficient R ≥ 0.5 or ≤ – 0.5 being considered positive and negative correlations, respectively), |log_2_ fold change|> 1, LDA score > 2 in LEfSe or adjusted *P*-value < 0.05 in DESeq2 were used in the present study. During the weaning, some ASVs and modules were associated with diarrhea, while some were associated with non-diarrhea (Fig. 4 and Fig. S2).

With the three levels of evaluation criteria, the identified diarrhea-associated ASVs were aggregated in *Bacteroides* (ASV39, ASV41, ASV145, ASV170, ASV206, and ASV259)*.* These members might be biomarkers of diarrhea risk. Muribaculaceae (ASV28 and ASV44)*, UBA1819* (ASV151), *Barnesiella* (ASV497)*,* and *Ruminococcaceae_UCG-005* (ASV254) were non-diarrhea-associated ASVs in the co-occurrence pattern, and ASV28 and ASV497 had a direct inhibitory relationship with the members of D-M1 (Fig. 4A). Muribaculaceae, which was previously assigned as family S24-7 or Homeothermaceae, is a common and abundant family of symbiotic bacteria in the gut and specialized in fermenting complex carbohydrates [67]. It responded most positively to acarbose treatment for diabetes [68] and was linked to longevity [69]. *Barnesiella* belongs to the family Porphyromonadaceae within the phylum Bacteroidota. It was found to suppress the growth of intestinal vancomycin-resistant *Enterococcus* [70]. Members of Ruminococcaceae are mostly butyrate-producing bacteria. Weese et al. suggested that Firmicutes (particularly Lachnospiraceae and Ruminococcaceae)/Proteobacteria ratio might be used to potentially predict and prevent colic [71]. Although some members of Ruminococcacea were associated with diarrhea, both ASV151 and ASV254 (both assigned to Ruminococcaceae) were associated with non-diarrhea. So Muribaculaceae (ASV28 and ASV44), *UBA1819* (ASV151), *Barnesiella* (ASV497)*,* and *Ruminococcaceae_UCG-005* (ASV254) might be used as potential probiotics for this specific commercial farm, with this specific microbial colonization patterns. The sequences of these ASVs were included in Table 2, and these ASVs can be verified in future studies. Fusobacteriaceae dominated D-M2 (Fig. 4C). Fusobacteriaceae was reported to have high relative abundances in dairy calves suffering from diarrhea, either infected [72] or uninfected [65], which indicates that module analysis can help identify bacteria associated with diarrhea or otherwise. With this module analysis, Muribaculaceae and *Prevotella* were identified as the core microbiota resisting diarrhea in weaning calves. Kim et al. reported that substituting fermented soybean meal (FSBM) for soybean meal (SBM) at 5% level in calf starter reduced the incidence of diarrhea and improved immunocompetence in neonatal calves after microbial infection [73]. The reason for the positive effects of FSBM on immunocompetence was not reported, but in a recent study, Feizi et al. found that FSBM increased the abundance of *Prevotella ruminicola* in the rumen of dairy calves [74]. Essential oils showed similar effects on dairy calves, increasing the Prevotellaceae abundance in the rumen [75] and decreasing the morbidity of neonatal diarrhea among pre-weaning calves [76]. Furthermore, a recent study reported that sodium humate and glutamine in combination also elevated the abundance of *P. ruminicola* in the rectum while reducing diarrhea incidence among dairy calves during the weaning period [42]. Tap et al. reported that Prevotellaceae enterotype was less susceptible to irritable bowel syndrome (IBS) compared with Bacteroidaceae enterotype [77]. Therefore, future research is warranted to investigate the relationship between calf diarrhea and *Prevotella* as a genus and its species. *Prevotella* may also be explored for its preventative ability to reduce calf diarrhea.

**Table 2:** The sequences of ASVs with potentials being probiotics and biomarker of diarrhea risk

ASV ID	Taxonomy	Sequence
ASV4	*Prevotella*_*2*	TGAGGAATATTGGTCAATGGACGGGAGTCTGAACCAGCCAAGTAGCGTGCAGGATGACGGCCCTATGGGTTGTAAACTGCTTTTATAGGGGGATAAAGTGTGCCACGTGTGGCATATTGCAGGTACCCTATGAATAAGGACCGGCTAATTCCGTGCCAGCAGCCGCGGTAATACGGAAGGTCCGGGCGTTATCCGGATTTATTGGGTTTAAAGGGAGCGTAGGCCGTCTTATAAGCGTGTTGTGAAATGTCGGGGCTCAACCTGGGCATTGCAGCGCGAACTGTGAGACTTGAGTGCGCAGGAAGTAGGCGGAATTCGTCGTGTAGCGGTGAAATGCTTAGATATGACGAAGAACTCCGATTGCGAAGGCAGCCTGCTGTAGCGCAACTGACGCTGAAGCTCGAAAGCGTGGGTATCGAACAGG
ASV14	*Alloprevotella*	TGAGGAATATTGGTCAATGGACGCAAGTCTGAACCAGCCAAGTAGCGTGCAGGACGACGGCCCTCCGGGTTGTAAACTGCTTTTAGTTGGGAATAAAGTGCAGCTCGTGAGCTGTTTTGTATGTACCATCAGAAAAAGGACCGGCTAATTCCGTGCCAGCAGCCGCGGTAATACGGAAGGTCCGGGCGTTATCCGGATTTATTGGGTTTAAAGGGAGCGCAGGCGGACTCTTAAGTCAGTTGTGAAATACGGCGGCTCAACCGTCGGACTGCAGTTGATACTGGGAGTCTTGAGTGCACACAGGGATGCTGGAATTCATGGTGTAGCGGTGAAATGCTCAGATATCATGAAGAACTCCGATCGCGAAGGCAGGTATCCGGGGTGCAACTGACGCTGAGGCTCGAAAGTGCGGGTATCAAACAGG
ASV26	*Prevotella*_*2*	TGAGGAATATTGGTCAATGGACGAGAGTCTGAACCAGCCAAGTAGCGTGCAGGACGACGGCCCTATGGGTTGTAAACTGCTTTTATAGGGGGATAAAGTGTGCCACGTGTGGCATATTGCAGGTACCCTATGAATAAGGACCGGCTAATTCCGTGCCAGCAGCCGCGGTAATACGGAAGGTCCGGGCGTTATCCGGATTTATTGGGTTTAAAGGGAGCGTAGGCCGTCTTATAAGCGTGTTGTGAAATGTCGGGGCTCAACCTGGGCATTGCAGCGCGAACTGTGAGACTTGAGTGCGCAGGAAGTAGGCGGAATTCGTCGTGTAGCGGTGAAATGCTTAGATATGACGAAGAACTCCGATTGCGAAGGCAGCCTGCTGTAGCGCAACTGACGCTGAAGCTCGAAAGCGTGGGTATCGAACAGG
ASV43	*Prevotella*_*9*	TGAGGAATATTGGTCAATGGACGAGAGTCTGAACCAGCCAAGTAGCGTGCAGGAAGACGGCCCTATGGGTTGTAAACTGCTTTTATAAGGGAATAAAGTGAGTCTCGTGAGACTTTTTGCATGTACCTTATGAATAAGGACCGGCTAATTCCGTGCCAGCAGCCGCGGTAATACGGAAGGTCCGGGCGTTATCCGGATTTATTGGGTTTAAAGGGAGCGTAGGCCGGAGATTAAGCGTGTTGTGAAATGTAGACGCTCAACGTCTGCACTGCAGCGCGAACTGGTTTCCTTGAGTACGCACAAAGTGGGCGGAATTCGTGGTGTAGCGGTGAAATGCTTAGATATCACGAAGAACTCCGATTGCGAAGGCAGCTCACTGGAGCGCAACTGACGCTGAAGCTCGAAAGTGCGGGTATCGAACAGG
ASV28	Muribaculaceae	TGAGGAATATTGGTCAATGGGCGCAGGCCTGAACCAGCCAAGTCGCGTGAGGGAGGACGGTCCTACGGATTGTAAACCTCTTTTGTCGGGGAGTAACGTGCGGGACGCGTCCCGTATTGAGAGTACCCGAAGAAAAAGCATCGGCTAACTCCGTGCCAGCAGCCGCGGTAATACGGAGGATGCGAGCGTTATCCGGATTTATTGGGTTTAAAGGGTGCGCAGGCGGCGCGCCAAGTCAGCGGTCAAAGTTCCGGGCTCAACCCGGTGTCGCCGTTGAAACTGGCGTGCTCGAGTGCGTGCGAGGAAGGCGGAATGCGTTGTGTAGCGGTGAAATGCATAGATATGACGCAGAACTCCGATTGCGAAGGCAGCTTTCCAGCGCGCTACTGACGCTGAGGCACGAAAGCGTGGGGATCGAACAGG
ASV44	Muribaculaceae	TGAGGAATATTGGTCAATGGGCGCAGGCCTGAACCAGCCAAGTCGCGTGAGGGAAGACGGTCCTACGGATTGTAAACCTCTTTTGTCGGGGAGTAACGTGCGGGACGCGTCCCGTATTGAGAGTACCCGAAGAAAAAGCATCGGCTAACTCCGTGCCAGCAGCCGCGGTAATACGGAGGATGCGAGCGTTATCCGGATTTATTGGGTTTAAAGGGTGCGCAGGCGGCGCGCCAAGTCAGCGGTCAAAGTTCCGGGCTCAACCCGGTGTCGCCGTTGAAACTGGCGTGCTCGAGTGCGTGCGAGGAAGGCGGAATGCGTTGTGTAGCGGTGAAATGCATAGATATGACGCAGAACTCCGATTGCGAAGGCAGCTTTCCAGCGCGCTACTGACGCTGAGGCACGAAAGCGTGGGGATCGAACAGG
ASV151	*UBA1819*	TGGGGAATATTGCACAATGGGGGAAACCCTGATGCAGCGACGCCGCGTGGAGGAAGAAGGTTTTCGGATTGTAAACTCCTGTCTTCGGGGACGATAATGACGGTACCCGAGGAGGAAGCCACGGCTAACTACGTGCCAGCAGCCGCGGTAAAACGTAGGTGGCAAGCGTTGTCCGGAATTACTGGGTGTAAAGGGAGCGCAGGCGGGTCGGCAAGTTGGAGGTGAAAGCTGTGGGCTCAACCCACAAACTGCCTTCAAAACTGCCGATCTTGAGTGGTGTAGAGGTAGGCGGAATTCCCGGTGTAGCGGTGGAATGCGTAGATATCGGGAGGAACACCAGTGGCGAAGGCGGCCTACTGGGCACTAACTGACGCTGAGGCTCGAAAGCATGGGTAGCAAACAGG
ASV497	*Barnesiella*	TGAGGAATATTGGTCAATGGTCGGCAGACTGAACCAGCCAAGTCGCGTGAGGGAAGACGGCCCTACGGGTTGTAAACCTCTTTTGTCGGAGAGTAAAGTACGCTACGTGTAGTGTATTGCAAGTATCCGAAGAAAAAGCATCGGCTAACTCCGTGCCAGCAGCCGCGGTAATACGGAGGATGCAAGCGTTATCCGGATTTATTGGGTTTAAAGGGTGCGTAGGCGGCACGCCAAGTCAGCGGTGAAATTTCCGGGCTCAACCCGGACTGTGCCGTTGAAACTGGCGAGCTAGAGTACACAAGAGGCAGGCGGAATGCGTGGTGTAGCGGTGAAATGCATAGATATCACGCAGAACCCCGATTGCGAAGGCAGCCTGCTAGGGTGAAACAGACGCTGAGGCACGAAAGCGTGGGTATCGAACAGG
ASV254	*Ruminococcaceae UCG-005*	TGGGGAATATTGGGCAATGGGGGAAACCCTGACCCAGCAACGCCGCGTGAAGGAAGAAGGCCCTCGGGTTGTAAACTTCTTTTACCAGGGACGAAGGACGTGACGGTACCTGGAGAAAAAGCAACGGCTAACTACGTGCCAGCAGCCGCGGTAATACGTAGGTTGCAAGCGTTGTCCGGATTTACTGGGTGTAAAGGGCGTGTAGGCGGAGCTGCAAGTCAGATGTGAAATCCCGGGGCTCAACCCCGGAACTGCATTTGAAACTGTAGCCCTTGAGTATCGGAGAGGCAAGCGGAATTCCTAGTGTAGCGGTGAAATGCGTAGATATTAGGAGGAACACCAGTGGCGAAGGCGGCTTGCTGGACGACAACTGACGCTGAGGCGCGAAAGCGTGGGGAGCAAACAGG
ASV48	*Clostridium sensu_stricto_1*	TGGGGAATATTGCACAATGGGGGAAACCCTGATGCAGCAACGCCGCGTGAGTGATGACGGCCTTCGGGTTGTAAAGCTCTGTCTTTGGGGACGATAATGACGGTACCCAAGGAGGAAGCCACGGCTAACTACGTGCCAGCAGCCGCGGTAATACGTAGGTGGCAAGCGTTGTCCGGATTTACTGGGCGTAAAGGGAGCGTAGGCGGATTTTTAAGTGGGATGTGAAATACCCGGGCTCAACCTGGGTGCTGCATTCCAAACTGGAAATCTAGAGTGCAGGAGGGGAAAGTGGAATTCCTAGTGTAGCGGTGAAATGCGTAGAGATTAGGAAGAACACCAGTGGCGAAGGCGACTTTCTGGACTGTAACTGACGCTGAGGCTCGAAAGCGTGGGGAGCAAACAGG
ASV39	*Bacteroides*	TGAGGAATATTGGTCAATGGACGAGAGTCTGAACCAGCCAAGTAGCGTGAAGGATGAAGGTCCTACGGATTGTAAACTTCTTTTATAAGGGAATAAACCCTCCCACGTGTGGGAGCTTGTATGTACCTTATGAATAAGCATCGGCTAACTCCGTGCCAGCAGCCGCGGTAATACGGAGGATGCGAGCGTTATCCGGATTTATTGGGTTTAAAGGGAGCGCAGACGGGTCGTTAAGTCAGCTGTGAAAGTTTGGGGCTCAACCTTAAAATTGCAGTTGATACTGGCGTCCTTGAGTGCGGTTGAGGTGTGCGGAATTCGTGGTGTAGCGGTGAAATGCTTAGATATCACGAAGAACTCCGATTGCGAAGGCAGCACACTAAGCCGTAACTGACGTTCATGCTCGAAAGTGTGGGTATCAAACAGG
ASV41	*Bacteroides*	TGAGGAATATTGGTCAATGGGCGAGAGCCTGAACCAGCCAAGTAGCGTGAAGGATGAAGGTCCTACGGATTGTAAACTTCTTTTATAAGGGAATAAAACGCTCCACGTGTGGAGCCTTGTATGTACCTTATGAATAAGCATCGGCTAACTCCGTGCCAGCAGCCGCGGTAATACGGAGGATGCGAGCGTTATCCGGATTTATTGGGTTTAAAGGGAGCGCAGACGGGATGTTAAGTCAGCTGTGAAAGTTTGCGGCTCAACCGTAAAATTGCAGTTGATACTGGCGTTCTTGAGTGCAGTTGAGGTGTGCGGAATTCGTGGTGTAGCGGTGAAATGCTTAGATATCACGAAGAACTCCGATTGCGAAGGCAGCTCACTAAACTGTAACTGACGTTCATGCTCGAAAGTGTGGGTATCAAACAGG
ASV145	*Bacteroides*	TGAGGAATATTGGTCAATGGGCGAGAGCCTGAACCAGCCAAGTAGCGTGAAGGATGAAGGTCCTATGGATTGTAAACTTCTTTTATAAGGGAATAAAACGCTCCACGTGTGGAGCCTTGTATGTACCTTATGAATAAGCATCGGCTAACTCCGTGCCAGCAGCCGCGGTAATACGGAGGATGCGAGCGTTATCCGGATTTATTGGGTTTAAAGGGAGCGCAGACGGGATGTTAAGTCAGCTGTGAAAGTTTGCGGCTCAACCGTAAAATTGCAGTTGATACTGGCGTTCTTGAGTGCAGTTGAGGTGTGCGGAATTCGTGGTGTAGCGGTGAAATGCTTAGATATCACGAAGAACTCCGATTGCGAAGGCAGCTCACTAAACTGTAACTGACGTTCATGCTCGAAAGTGTGGGTATCAAACAGG
ASV170	*Bacteroides*	TGAGGAATATTGGTCAATGGTCGGAAGACTGAACCAGCCAAGTAGCGTGAAGGATGAAGGTTCTATGGATTGTAAACTTCTTTTATACGGGAATAAAACCACCTACGTGTAGGTGCTTGTATGTACCGTATGAATAAGCATCGGCTAACTCCGTGCCAGCAGCCGCGGTAATACGGAGGATGCGAGCGTTATCCGGATTTATTGGGTTTAAAGGGAGCGTAGACGGGGGATTAAGTCAGTTGTGAAAGGCTGCGGCTCAACCGCAGCACTGCAGTTGATACTGGTTTCCTTGAGTGCGGTTGAGGTGTATGGAATTCGTGGTGTAGCGGTGAAATGCTTAGATATCACGAAGAACTCCGATTGCGAAGGCAGTACACTAAGCCGTAACTGACGTTGAGGCTCGAAAGTGTGGGTATCAAACAGG
ASV206	*Bacteroides*	TGAGGAATATTGGTCAATGGCCGGAAGGCTGAACCAGCCAAGTAGCGTGAAGGATGAAGGTTCTATGGATTGTAAACTTCTTTTATACGGGAATAAAACCACCTACGTGTAGGTGCTTGTATGTACCGTATGAATAAGCATCGGCTAACTCCGTGCCAGCAGCCGCGGTAATACGGAGGATGCGAGCGTTATCCGGATTTATTGGGTTTAAAGGGAGCGTAGACGGGATGTTAAGTCAGTTGTGAAAGGCTGCGGCTCAACCGCAGCACTGCAGTTGATACTGGCGTCCTTGAGTGCGGTTGAGGTATGTGGAATTCGTGGTGTAGCGGTGAAATGCTTAGATATCACGAAGAACTCCGATTGCGAAGGCAGCATACTAAGCCGCTACTGACGTTGAGGCTCGAAAGTGTGGGTATCAAACAGG
ASV259	*Bacteroides*	TGAGGAATATTGGTCAATGGACGAGAGTCTGAACCAGCCAAGTAGCGTGAAGGATGAAGGTCCTACGGATTGTAAACTTCTTTTATAAGGGAATAAAACCTCCCACGTGTGGGAGCTTGTATGTACCTTATGAATAAGCATCGGCTAACTCCGTGCCAGCAGCCGCGGTAATACGGAGGATGCGAGCGTTATCCGGATTTATTGGGTTTAAAGGGAGCGCAGACGGGATGTTAAGTCAGCTGTGAAAGTTTGCGGCTCAACCGTAAAATTGCAGTTGATACTGGCGTTCTTGAGTGCAGTTGAGGTGTGCGGAATTCGTGGTGTAGCGGTGAAATGCTTAGATATCACGAAGAACTCCGATTGCGAAGGCAGCTCACTAAACTGTAACTGACGTTCATGCTCGAAAGTGTGGGTATCAAACAGG

Consistent changes in relative abundance of *Bacteroides*, *Butyricicoccus*, *Faecalibacterium*, *Alloprevotella*, and *Fusobacterium* were observed in both trial 1 and trial 2 (Fig. 1, 2 and 6). When the fecal microbiota was examined in detail as the calves transitioned from pre-diarrhea to diarrhea and then to post-diarrhea phases in trial 2, the peak of both *Clostridium_sensu_stricto_1* and *Fusobacterium* coincided with the peak of diarrhea. It has been reported that *Clostridium_sensu_stricto_1* might cause epithelial inflammation in piglets [78] and stunting in infants (defined as height-for-age Z score equal to or lower than – 2, [79]). Therefore, research is needed to further investigate these two genera with respect to their role in calf diarrhea. Our co-occurrence analysis (Fig. 7) showed that the post-D-M1 might be a driver of diarrhea recovery because of the close interaction between its constituent members and the inhibitory relationship with D-P-M. *Prevotella*_*2* and *Alloprevotella* increased their relative abundance in the post-diarrhea phase (Fig. 6C), and they dominated the post-D-M1 (Fig. 7F), which supports the importance of Prevotellaceae in resisting calf diarrhea. In particular, *Prevotella*_*2* (ASV4 and ASV26), *Alloprevotella* (ASV14) and *Prevotella*_*9* (ASV43), their sequences were included in Table 2, might be potential probiotics for preventing diarrhea in early stage. It should be noted that although most of the constituent members of post-D-M1 were detected before diarrhea (module 3 and 4 in Fig. 7A), their betweenness and close centrality increased in post-D-M1. This suggests that the interactions among different bacteria might play an important role in maintaining intestinal homeostasis in the gut. These potential probiotics may be supplemented in the first week after birth to prevent diarrhea, and fiber diets [80] or FSBM [71] may improve their efficacy. *Clostridium_sensu_stricto_1* (ASV48, Fig. 7, Table 2), was negatively related with *Prevotella*_*2* (ASV4 and ASV26), *Alloprevotella* (ASV14) and *Prevotella*_*9* (ASV43). All of the four ASVs established the negative relationship between the post-D-M1 and D-P-M. Thus, *Clostridium_sensu_stricto_1* (ASV48) might be a biomarker of diarrhea risk in the early stage.

## Conclusions

In conclusion, microbial successions of the gut microbiome in dairy calves were rapid, and daily sampling is needed to capture the rapid dynamic gut microbial successions. Promoting indigenous *Prevotella* and Muribaculaceae might be a new strategy to reduce the incidence of diarrhea in neonatal calves and help calves to go through the weaning transition smoothly. *Prevotella_2* (ASV4 and ASV26), *Prevotella_9* (ASV43), *Alloprevotella* (AVS14), unclassified Muribaculaceae (ASV28 and ASV44), *UBA1819* (ASV151), *Ruminococcaceae_UCG-005* (ASV254), and *Barnesiella* (ASV497) might be used as probiotics to reduce or prevent calf diarrhea; *Clostridium_sensu_stricto_1* (ASV48) might be a useful biomarker of diarrhea risk in this large-scale dairy farm locating in subtropical monsoon climate zone with automated milk feeder system.

## Supplementary Information

**Additional file 1:  Fig. S1.** A schematic showing the experimental design, milk feeding, and fecal sample collection of the two trials.

**Additional file 2: Fig. S2.** Heatmap of the ASVs associated with diarrheic status in stage 4 of trial 1. The ASVs were identified based on fold change and analysis using LEfSe or DESeq2. 

**Additional file 3: Table S1.** The ASVs in co-occurrence pattern of trial 1 stage 4.

**Additional file 4: Fig. S3. **Principal coordinates analysis (PCoA) plot of fecal microbiotas among ages in trial 2. The fecal microbiotas differences (P values) between diarrheic and non-diarrheic calves within the same age were compared.

**Additional file 5: Fig. S4. **Heatmap of the ASVs that identified to be diarrheic status transition-associated in trial 2. The ASVs were identified based on fold change and analysis using LEfSe or DESeq2.

## Data Availability

The raw sequencing data generated in this study are publicly available in NCBI Sequence Read Archive (http://www.ncbi.nim.nih.gov/sra) under the accession number PRJNA716761.

## Abbreviations

*E. coli* K99^+^: *Escherichia coli* K99^+^
BRV: Bovine rotavirus
BCoV: Bovine coronavirus
ASV: Amplicon sequence variant
Faith’s PD: Faith’s phylogenetic diversity
ANOSIM: Aanalysis of similarity
PCoA: Principal coordinates analysis
PERMANOVA: Permutational multivariate analysis of variance
LEfSe: Linear discriminant analysis Effect Size
D-M1: Diarrhea module 1
D-M2: Diarrhea module 2
ND-M: Non-diarrhea module
pre-D-M: Pre-diarrhea phase-associated module
D-P-M: Diarrhea phase-associated module
post-D-M1/2: Post-diarrhea phase-associated modules
