# Supplementary figures and images for: Fecal microbiota dynamics and its relationship to diarrhea and health in dairy calves
Source: J Anim Sci Biotechnol. 2022 Oct 28;13:132. doi: 10.1186/s40104-022-00758-4 (PMC9616619; doi:10.1186/s40104-022-00758-4)

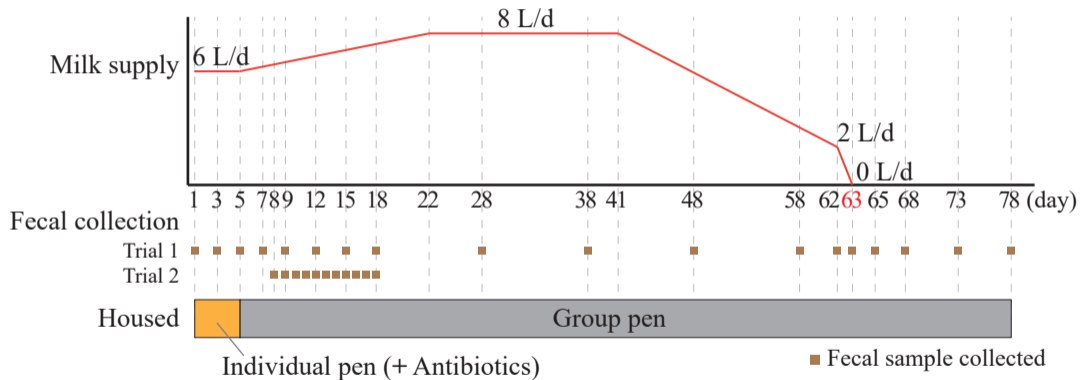

Supplement: Supplementary file 1 — Additional file 1: Fig. S1. A schematic showing the experimental design, milk feeding, and fecal sample collection of the two trials. [file 40104_2022_758_MOESM1_ESM.pdf]

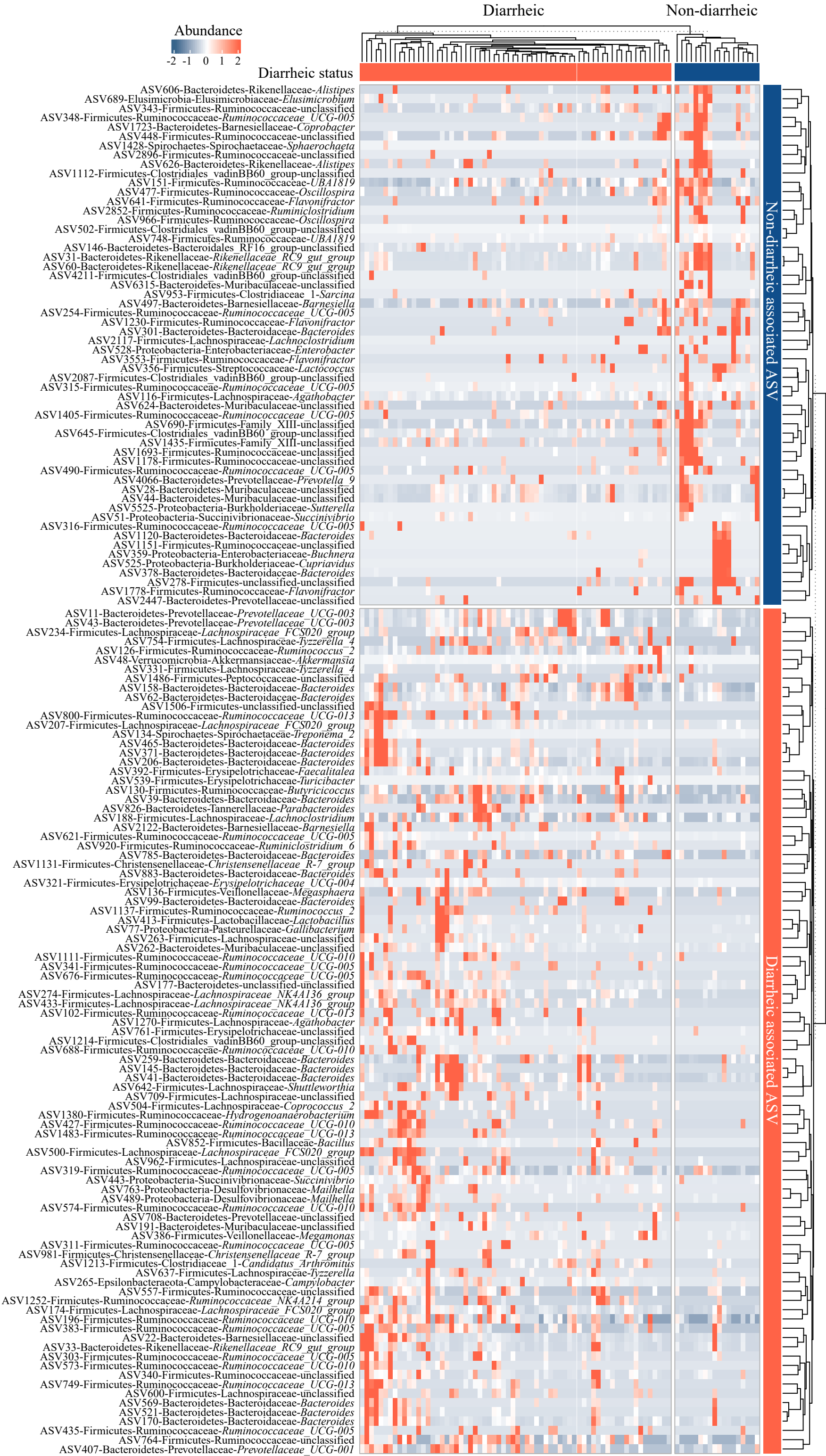

Supplement: Supplementary file 2 — Additional file 2: Fig. S2. Heatmap of the ASVs associated with diarrheic status in stage 4 of trial 1. The ASVs were identified based on fold change and analysis using LEfSe or DESeq2. [file 40104_2022_758_MOESM2_ESM.pdf]

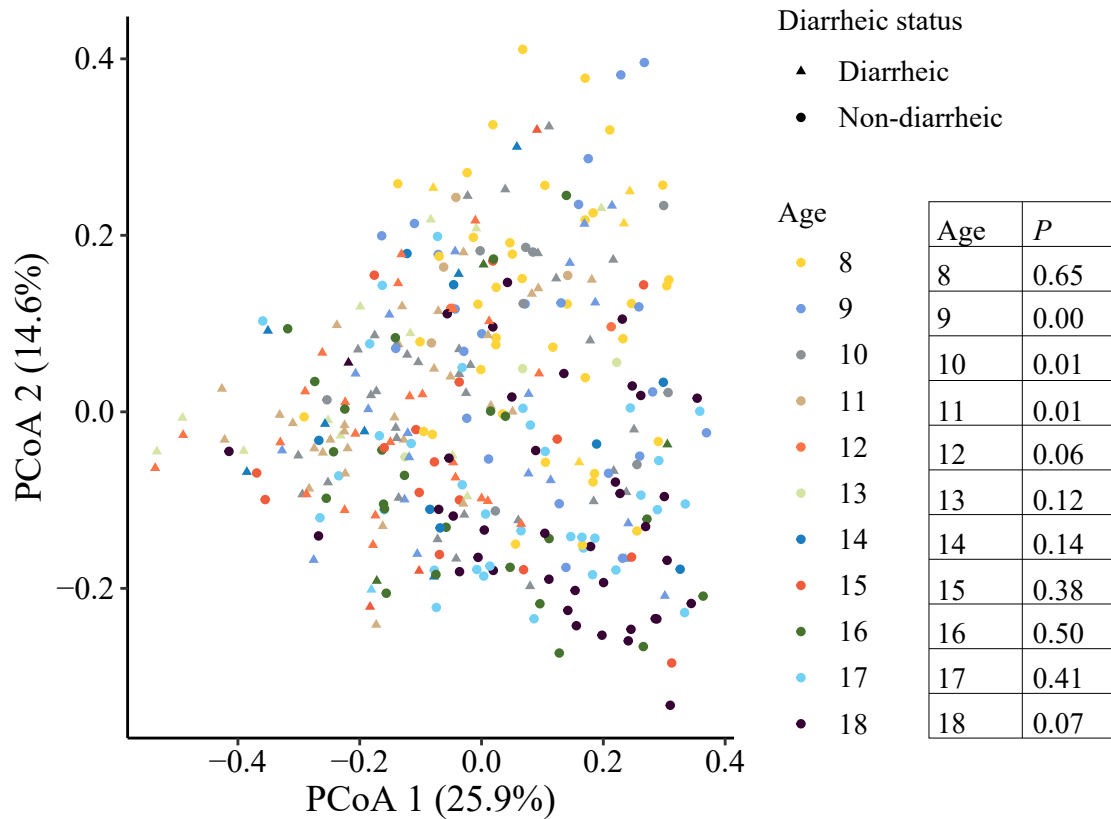

Supplement: Supplementary file 4 — Additional file 4: Fig. S3. Principal coordinates analysis (PCoA) plot of fecal microbiotas among ages in trial 2. The fecal microbiotas differences (P values) between diarrheic and non-diarrheic calves within the same age were compared. [file 40104_2022_758_MOESM4_ESM.pdf]

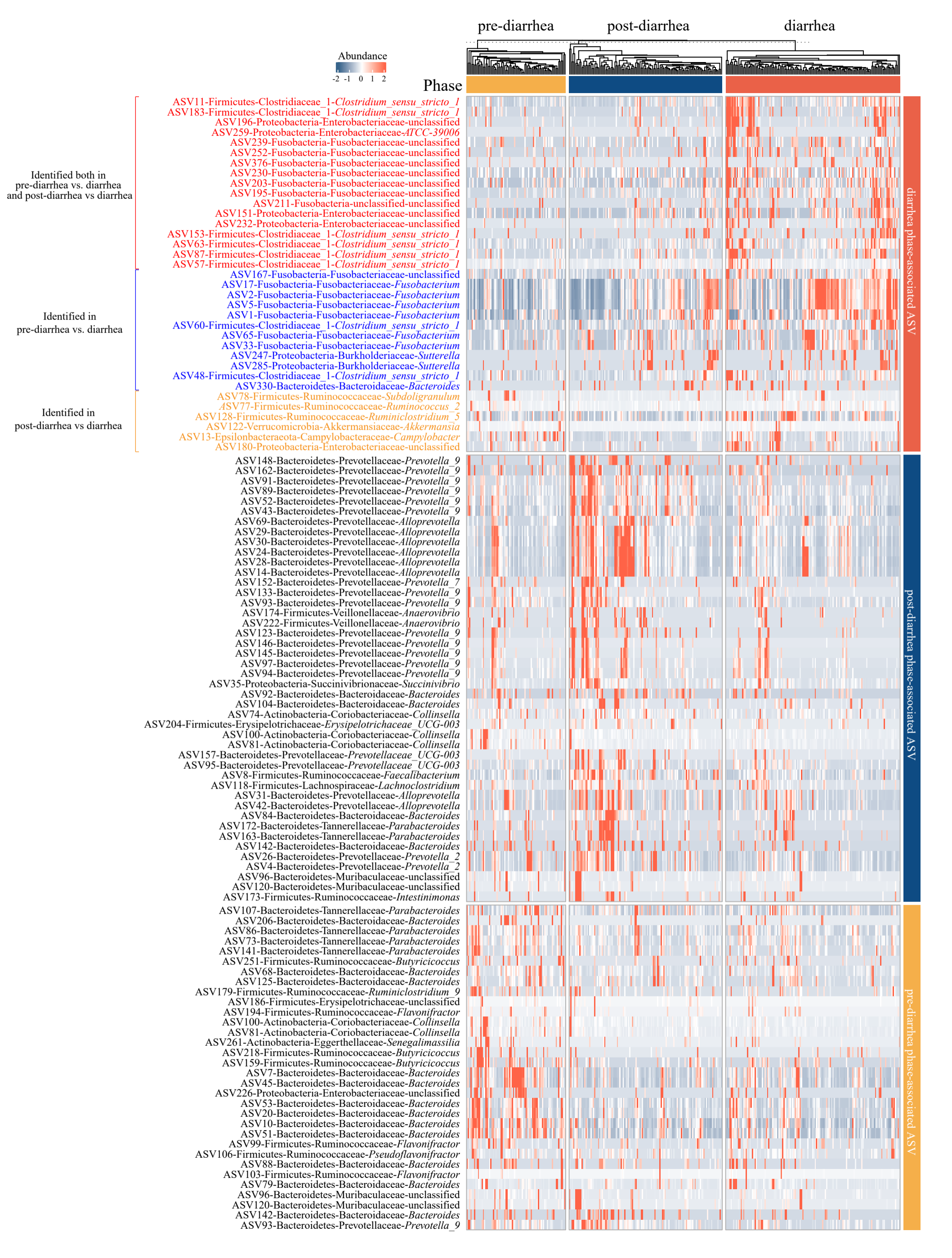

Supplement: Supplementary file 5 — Additional file 5: Fig. S4. Heatmap of the ASVs that identified to be diarrheic status transition-associated in trial 2. The ASVs were identified based on fold change and analysis using LEfSe or DESeq2. [file 40104_2022_758_MOESM5_ESM.pdf]
